# Debate on Tracheotomy in Epilepsy at the Medical Society of London

**Published:** 1853-07

**Authors:** 


*Debate on Tracheotomy in Epilepsy at the Medical Society of London.*—Dr. Radcliffe read a paper "*On the Questionable Utility of Tracheotomy in the Treatment of any kind of Epilepsy.*"—In order to arrive at the object of his paper, the author depended chiefly upon a critical examination of the cases of epilepsy in which tracheotomy has been practised, and to this examination he at once proceeded :

*Mr. Cane's Case.*—The patient was a boatman, aged twenty-four, who had been epileptic for seven or eight years. The fits were severe and frequent. The operation was performed during a fit, in consequence of a state of asphyxial-coma that had lasted nineteen hours. The relief was immediate, and no fits have followed the operation. The habits of the patient were very irregular and intemperate, and he was discharged from his employment on this account about ten months ago. The tube is still worn, and curiously enough, it is worn with a cork in the opening.

*Mr. Anderson's Case.*—The patient in this case was a stout, thick-set, muscular female, aged thirty-six, the daughter of an epileptic father, and herself epileptic for twenty-four years. Her complexion was ruined by the former use of nitrate of silver. The operation was performed in March, 1851, and the tube was worn until her death, which happened in a fit about four months ago. After the operation the fits continued as before—possibly a little less frequently and severely, but decidedly of the same character. Her health and spirits also are said to have undergone some slight improvement, and she lost a numbness in the right arm which had previously distressed her, but those who knew her best doubt the existence of any appreciable change of this kind until about two or three months before her death—sixteen months after the operation. The following notes of the final seizure are from Mr. Anderson :

"Eight A. M. : Had been up and dressed ; heard to fall heavily. A woman removed the inner tube from the trachea as she was in a fit apparently more severe than usual. She 'snorted loudly ;' nails of a deeper color. She was placed on the bed, as the woman thought she would recover as usual." The woman here referred to says the patient was black in the face and violently convulsed, and that death must have taken place within ten minutes. The body was examined twenty-four hours after death, and the following are the particulars supplied by Mr. Anderson :—"Body extremely muscular ; cadaverous rigidity still present ; not much fat. Head : Vessels of scalp much congested ; skull thick, and dura mater so universally adherent that the skull-cap could not be removed until the dura mater was divided. The sinuses were filled with dark blood, and on the removal of the brain an unusual quantity of dark blood flowed from the spinal canal. On either side of the longitudinal sinus, and on the inner side of the frontal bone, two or three growths of bone were found, and to these the dura mater was so firmly adherent that, on attempting to separate it, it was torn through, and portions remained attached. The largest of the exostoses was about an inch and a half in circumference, and projected about half an inch from the surface of the bone. No alteration was observed in the corresponding portion of the cerebrum. The brain was softer than natural, and the puncta were more than usually distinct. There was little fluid in the ventricles, but the choroid plexuses were congested. Lungs: These organs were collapsed, occupying but little more than a third of the thoracic cavity, and somewhat congested at their posterior margin; structure healthy. Heart: Larger than usual (perhaps a fourth;) cavities, especially the left, distended with blood. It was surrounded with fat, and its structure flabby;* valves healthy. Liver, kidneys, and spleen: Highly congested. Uterus natural, but cysts containing viscid fluid in the ovaries. Small intestines (especially lower part of the ilium) congested, and the mesenteric glands enlarged. Internal jugular, above the level of the omohyoid, almost empty."

*Mr. Mackarsie's Case.*—R. W——, aged forty, and epileptic for twenty years. Latterly the fits had become much more frequent and severe, the subsequent torpor much prolonged, and the mind much impaired. His complexion had a congested, mahogany-like tint. Two years previously he had had two attacks of paralysis, but his present health, apart from the fits, is pretty good. Tracheotomy was performed on the 24th of August, 1852, by means of the tracheotome. On the day following the operation inflammatory action began in the lungs, and continued until the 6th of August. Thus,—August 25th: "A large quantity of mucus has passed from the tube." 26th: "The patient has been hot and feverish, and passed a restless night; tongue furred; pulse 100; a large quantity of mucus passing through the tube." 27th: "Pulse 100, full and hard." 28th: "Tongue still furred." 31st: "Violent hæmoptysis." Sept. 1st: "Violent return of hæmoptysis;" "left lung congested, dull on percussion, and respiratory murmur feeble." 2d: "Expectorates bloody mucus;" "dulness on percussion not so marked; respiratory murmur more audible." 3d: "Still bloody expectoration." 4th: "Pulse 90 and soft." 6th: "Pulse 75, soft; respiration free; dulness on percussion gone." Again, on the 20th September, and for some days afterwards, there was feverishness, attended with bilious vomiting, requiring salines, calomel, and prussic acid. The fits, however, kept away until the second week in October, when four or five slight ones happened. After this true fits made their appearance, and continued to recur with their usual frequency, though in a mitigated form, until about two months ago, when the tube was withdrawn by the patient's wife, (who throughout has been greatly opposed to, and dissatisfied with the operation,) since which time the fits are as bad as ever, and the mental condition worse than ever. Mr. Mackarsie is fully of opinion that during the time the tube was in the trachea the mind was more active, the complexion less congested, and the fits less severe.

*Mr. J. A. Lockhart Clarke's Case.*—In this case the patient was a female, twenty-three years of age, who had been epileptic for twelve years. The fits were very violent and very frequent. Laryngotomy, not tracheotomy, was performed about three months ago, and the tube worn until recently, when it was removed, in consequence of their being no perceptible alteration either in the frequency, or in the severity of the fits.

* Dr. Jenner examined a portion of this heart microscopically and found some slight degree of fatty degeneration.

*Mr. Henry Thompson's Case.*—The main facts of this case are substantially these : The patient was an epileptic of twenty years standing, whose intellect had suffered considerably. Tracheotomy was performed nearly three months ago. Before the operation the fits were frequent and violent, and the subsequent sopor prolonged ; since the operation the fits have altered little in frequency and violence, but the subsequent torpor is greatly abridged. The general health also is improved, and the mind much clearer than it was.

*Dr. Tyler Smith's Case.*—Sarah B——, the wife of a gamekeeper at Debden in Essex, and the mother of four children. She has been e[illegible]ileptic since puberty, and chiefly about the menstrual period. The numbers of the fits during the month were sometimes as many as twenty, but generally not more than five or six. The fits themselves were usually preceded by the scream and attended with much lividity of the head and neck; the convulsions were very violent and the subsequent sopor protracted. The mental state was one of great inanity. There had been several paroxysms of insanity, and twice the patient had been in a lunatic asylum. During the month that she remained in the hospital before the operation there were nine fits; during the month after the operation there were five fits. The operation itself was performed on the 13th of February by Mr. Lane. On the 15th, 16th and 17th she was restless, wakeful and unruly, with heat of skin, raised pulse, and furred tongue. She threw a glass at the nurse, and persisted in attempting to withdraw the tube from the neck, and her state required constant watching. Three weeks after this she was greatly depressed, her pulse feeble and wretched, her countenance anxious, and much viscid, fœtid phlegm passed from the tube; and this state continued for the greater part of a week. Since this time she has rallied, and now her mental condition is much better than it was during the month before the operation; her fits also are much better, the period of sopor is somewhat abbreviated, and the cry is lost; but still the convulsion is violent, the venous turgescence of the head and neck considerable, though less than it was, and once at least the tongue has been bitten.

*Dr. Andrea Verga's Case.*—This case cannot strictly be classed with the former cases, for the operation was performed unintentionally, and by the patient himself; but in all other respects it fulfils the required predicaments. It was originally reported in one of the Lombard journals, and copied thence into an early number of Schmidt's *Jahrbuch* for 1852. The main particulars are the following : A. B.——, aged twenty-five, was admitted into the great hospital at Milan, with his throat cut and his genitals severely mutilated, in consequence of a determined attempt at suicide. Six months afterwards the wounds had healed, with the exception of a free fistulous opening in the trachea; but the fits and despondency had undergone no change. The breath passed freely in and out of the artificial opening, and the fits recurred with equal frequency and force whether that opening were closed or not. In this state he was removed to a madhouse, and there he remained for three years, when he died of tabes, the fistula continuing open and the fits unabated up to the end. After death the brain and skin were found congested and the bowels somewhat ulcerated.

*Comments.*—Such are the chemical data upon which as yet the remedial value of tracheotomy in epilepsy has to be tested, and the question is whether or not they realize Dr. Hall's expectations, and justify the comments which have been passed upon them.

What of Mr. Crane's case? Here undoubtedly the results seem most marked, but do they not prove too much? There are no fits whatever after the operation, and this is not to be expected even on Dr. Hall's own premises. Moreover, fits do happen in all the other cases, and in some of them very severe fits, and this fact gives a probabilty of at least seven to one that the fits in this case did not keep away in consequence of the operation. It is to be remembered also, that the wearing of the cork in the tracheal tube did in fact place the patient in the same predicament as that in which he was before the windpipe was opened. Why the fits kept away it is not necessary to inquire, for nothing is more certain than that epilipsy may suddenly disappear and keep away for a long time, without any apparent cause.

What of Mr. Anderson's case? Here the main questions are as to the character of the fits, the state of the general health, and the cause of death. Were the fits improved in character? Possibly, but not probably. Dr. M. Hall, in his lectures at the College of Physicians, allowed that a fit had followed very shortly after the operation, in which the tongue had been bitten. A Mrs. Dwellie, living in the adjoining garret to the patient's, and who frequently went to the patient's assistance when she heard the noise and struggle of the fit, states explicitly that the convulsions were as frequent and violent, and the subsequent sopor as prolonged, after the operation as before it. A Mrs. Smith, also, an aunt of the patient, who had known her from childhood, and who saw her several times a week during the whole of her life, makes the same statement. Miss Lewis, on the contrary, who lives on the first floor of the house in the garret of which the patient lived, thinks the fits, after the operation, were not so severe or frequent as before it; but why she thinks so is not very evident. She saw her in but few fits, and in none (there is reason to believe) from the commencement. Indeed it is to be understood that this witness was infirm and half-crippled, and often quite an invalid; that she had to be fetched from the top of the house, and then to mount up two flights of stairs before she could get to the place where the patient was; so that the fit must have been far from its commencement before she could see it. The last fit, also, which was evidently of great violence, is spoken of only as "apparently more severe than usual," showing that the ordinary fits were severe, and the patient was "expected to recover as usual," showing that death had occurred unexpectedly in what was regarded as an ordinary fit. Concerning the state of the general health there are two opinions. Miss Lewis says this was better; Mrs. Dwellie and Mrs. Smith say there was no perceptible improvement until within two or three months from her death, fifteen or sixteen months after the operation. The cause of death is very obscure. It could not be, however, from the strangulation of laryngismus, for the inner tube was removed at the beginning of the last fit, as it was in all the fits in which the patient was watched. Indeed there was never any neglect or mismanagement about the tube, (which reflects the highest credit on Mr. Anderson's mechanical ingenuity,) and the patient herself had so schooled herself to it that she could remove and cleanse it, and did so remove and cleanse it many times a day. The fatty state of the heart, as Dr. Hall supposes, might have had something to do with death, for death happened shortly after the commencement of the seizure; but, on the other hand, it is not to be forgotten that there was stertorous breathing, blackness and turgescence of the head and neck, with distended sinuses, distinct cerebral puncta, and other signs showing that death might have been caused by coma.

What of Mr. Mackarsie's case? Here it is not difficult to imagine that the pulmonary inflammation and the subsequent febrile action may have had something to do with the absence of the fits during the first two months after the operation, for inflammation and fever are not only uncongenial to, but incompatible with, epilepsy. This inflammation also, even after its cessation, may have had something to do with the amelioration of the fits, by acting derivatively in regard to that mischief in the brain, the existence of which is to be argued from the two former attacks of paralysis. The fact, however, is not to be doubted—that the fits were "mitigated" and the mental state ameliorated after the operation. This is undeniable. Still the fits were true fits, and not mere warnings, and there is little if any reason for supposing that they gave up the characters of epilepsia gravior for those of epilepsia mitior; nor is it clear that the mind was not invigorated by hope or some other psychical stimulus, and that the fits were not subdued by the mind thus invigorated, the tracheal tube all the while acting merely as a charm by which to propitiate hope and her allies; nor is it clear that any diminished sopor after the fit may not have been the consequence rather than the cause of the mental invigoration. Time must elapse before these doubts can be resolved, and in the meantime it must not be forgotten that the wife of the patient was opposed to and dissatisfied with the operation.

What of Mr. Clarke's case? Nothing favourable to the operation.

What of Mr. Henry Thompson's case? In this case the fits recur as frequently as before, but the subsequent sopor and intermediate stupor are greatly diminished. Still it is by no means certain or even probable that the fits after the operation were of the character of epilepsia mitior, or that the diminished sopor and stupor were not the consequences of faith in the operation rather than of the operation itself.

What of Dr. Tyler Smith's case? In this case it is more than improbable that the fits underwent that modification which they ought to have done, or that any improvement in the symptoms is really due to the operation. All the fits after the operation were certainly not of the type of epilepsia mitior, for the convulsions were severe, and once at least the tongue was bitten. It is doubtful also whether the fits were really less frequent. During the first month of hospital life there were, it is true, nine fits, but this was a time when the patient was exposed to the agitating publicity of a hospital ward, with the fear of an operation before her eyes. The usual number of fits during the month would also seem to be from five to six, though occasionally ranging so high as twenty, and these numbers correspond with the numbers after the operation. It is clear also that as yet little can be said about mental improvement after the operation, seeing that a paroxysm of insanity and a week of extreme mental inanity form a part of this period. This being the case, it is not necessary to speculate whether such improvement is psychically or somatically the result of the operation.

What of Dr. Andrea Verga's case? Possibly very little, but certainly nothing in favour of the operation.

On looking over these cases, therefore, one conclusion is inevitable—namely, that severe fits have followed the operation—fits in which the tongue has been bitten, and one fit in which death has happened. Almost uniformly the convulsion has been as bad as ever. In Mr. Anderson's, Mr. Clarke's, Mr. Mackarsie's, and possibly in Dr. Andrea Verga's cases, the sopor after the fit, and the torpor between the fits were unaffected; in Mr. Henry Thompson's, and possibly in Dr. Tyler Smith's cases, they were relieved, though *how* they were relieved remains a matter of doubt. As judged, therefore, by the results of the cases in which it has been practically tested, the utility of tracheotomy in epilepsy would seem to be extremely doubtful; so doubtful, indeed, as to render it a matter of paramount and imperative necessity to pause and ponder well upon the evidence before again resorting to it, and this all the more because it is by no means certain that the remedy is not more dangerous than the disease, and because the inevitable result of the operation is to convert the patient into a dumb, whistling wretch, whose every breath is an annoyance to himself and others. In order to do this it will be necessary to examine epileptics, whose windpipes are sound as well as those whose windpipes are not sound. It will be necessary to determine how much of the epileptic asphyxia depends upon spasmodic "setting" of the whole chest, and how far this "setting" will negative the results of an opening in the windpipe. It will be necessary to go to the root of the matter, and determine whether, apart from organic disease, the larynx does close spasmodically in epilepsy, and whether such closure can exist at the time of life when epilepsy happens. In the meantime the absence of any stridulous inspiration in epilepsy, such as is heard in laryngismus stridulus, in the hooping-cough of children, and in certain organic diseases of the larynx, would seem to be a serious, if not fatal objection to the idea of laryngismus in epilepsy. The age of epileptics—namely, youth and manhood—is also an objection to the same effect; for judging from the history of laryngismus stridulus and hooping cough, pure spasmodic closure of the larynx is usually confined to the period antecedent to that at which epilepsy commences; indeed, as a rule, laryngismus stridulus is an affection of teething, and hooping-cough looses its characteristic hoop before puberty. This deduction is also borne out by the results which follow the division of the laryngeal nerves in the lower animals, as dogs and cats; for in these experiments the young animal is immediately suffocated by the closure of the glottidean chink, whereas the old animal goes on breathing without any evident diminution in the current of air.

Dr. Crisp regretted the absence of Dr. Marshall Hall, and was disposed to regard with favour the operation proposed by that gentleman. He deemed the whole subject worthy of further inquiry. Many epileptic patients are anæmic, but others are plethoric.

Dr. Barnes believed the subject to be very important, and the discussion of it not premature. He gave credit to the author of the paper for having recited the cases with fairness and candour, but inferred that Dr. Radcliffe had misapprehended Dr. Hall's views on the subject. He affirmed that each of the cases recited had exhibited marked improvement after the operation. Mr. Cane's case had been very successful; he admitted that. Mr. Anderson's patient had not died from asphyxia; but he did not admit Dr. Radcliffe's explanation. Dr. Jenner had found the heart in a state of fatty regeneration—a physical condition likely to cause death. Laryngismus is not met with in every case of epilepsy, neither is its presence essential.

Dr. Winn stated that epilepsy is not essentially accompanied by an anæmic condition of system.

Dr. Tyler Smith, in reference to his own case, explained that the attack of mania after the operation was probably to be attributed to the influence of chloroform. The fits are slighter, and the condition of the mind is clearly improved. He believed laryngismus to be essential to the disease, and that it occurs in adults without the stridulous breathing, except that the peculiar cry is a modification of that symptom. Lividity of countenance may be induced by spasms of the muscles of the neck, without the occurrence of laryngismus or closure of the glottis. Tracheotomy is not a serious operation.

Mr. Dendy thought that Dr. Hall had not been hardly treated, and that the cases had been fairly recited by Dr. Radcliffe. Tracheotomy is proposed, not as a remedy for epilepsy, but to relieve one of its symptoms. It is not a dangerous operation; yet he doubted if medical men would themselves submit to it. Laryngismus is neither the essence nor the proximate cause of the disease, and when it occurs it may pass away without the exhibition of any remedy. It is not important to decide whether this disease be essentially anæmic or not, but the case must be treated according to the nature of the patient's constitution. Congestion is not always productive of asphyxia, and may be relieved in some cases without the operation, or indeed without any remedy whatever. He believed that the good effects of the operation had been exaggerated.

Mr. H. Thompson, on referring to his own case, believed that the nature of the fit had been well made out, and stated that the family were unanimous in asserting that much benefit had followed the operation. The mind is clearly improved. He had witnessed the occurrence of a fit that morning, and found that loss of consciousness continued but during three or four minutes, instead of four hours, as had usually been the case previous to the operation. On that occasion the tube was not in a clean condition, and the same fact had been noticed on the occurrence of all the fits subsequent to the operation. He had removed the tube, and observed that respiration appeared to be suspended for a few seconds, no sound indicating the passage of air through the trachea. Mr. Bullock described the present improved condition of Dr. Tyler Smith's patient.

Dr. Camps approved the operation under the circumstances selected by Dr. Marshall Hall. He considered that an attack of epilepsy is not antagonized by existing inflammation or fever.

Mr. C. Clark mentioned a case which he had relieved by artificial respiration.

Dr. Radcliffe, in reply, said that he had taken up the question under consideration, not because it clashed with any of his own opinions respecting convulsive diseases,—which it did not,—but simply as a matter of fact that from its importance ought not to be passed over any longer. He had frankly expressed his present convictions, but he was perfectly willing and ready to change them whenever they were shown to be wrong. He had, he trusted, acted with all honor and sincerity, and his only regret was that Dr. Marshall Hall, (whom he and all the profession highly honored) had not been present in person to hear and reply to what he had ventured to say. In reply to an observation that had been made in the discussion, he would only say that he could not understand how the epileptic cry would be a proof of laryngismus.—*London Lancet*, May 14th, 1853.

